# Embracing telepharmacy: Unveiling Malaysians’ perceptions and knowledge through online survey

**DOI:** 10.1371/journal.pone.0307897

**Published:** 2024-08-27

**Authors:** Azlina Ahmad, Shubashini Gnanasan, Mahmathi Karuppannan

**Affiliations:** 1 School of Pharmacy, KPJ Healthcare University, Kota Seriemas, Nilai, Negeri Sembilan; 2 Department of Pharmacy Practice and Clinical Pharmacy, Faculty of Pharmacy, Universiti Teknologi MARA (UiTM) Selangor Branch, Puncak Alam Campus, Bandar Puncak Alam, Selangor, Malaysia; 3 Cardiology Therapeutics Research Group, Faculty of Pharmacy, Universiti Teknologi MARA (UiTM) Selangor Branch, Puncak Alam Campus, Selangor, Malaysia; Tehran University of Medical Sciences, ISLAMIC REPUBLIC OF IRAN

## Abstract

The expansion of information technologies, particularly during the COVID-19 pandemic, has notably increased the use of remote services, including telehealth. Telepharmacy, a subset of telehealth, offers remote pharmaceutical care services, benefiting patients by providing advice and consultations without the need for physical pharmacy visits. This study aimed to assess public perceptions and awareness of telepharmacy in Malaysia. A cross-sectional study was conducted from Nov 2022 to May 2023, involving 387 Malaysian citizens aged 18 and above. Data collection utilised Google Forms distributed via social medias, covering demographics, technological readiness, awareness, perceptions, and willingness related to telepharmacy. The study demonstrated high digital readiness among respondents, owning smartphones and being adept in utilising various digital features. However, there was a lack of awareness regarding the concept of telepharmacy. Despite predominantly positive perceptions of its potential, only 48.1% of respondents showed willingness to utilise telepharmacy services. While respondents exhibited readiness for digital engagement, there was a notable deficit in understanding telepharmacy. Though perceptions were positive, willingness to embrace telepharmacy was moderate. Addressing the knowledge gap through targeted education initiatives might enhance acceptance. Future research should focus on integrating telepharmacy into healthcare systems considering public preferences, thereby evaluating its actual implementation and outcomes among diverse demographics.

## Introduction

The use of information technologies has rapidly expanded over the years, especially during the pandemic of coronavirus disease (COVID-19). These technologies have been instrumental in facilitating remote work, online education, and telehealth services. One area that has seen significant growth is telepharmacy, which involves the remote provision of pharmaceutical care services. Telepharmacy, a branch of telehealth, allows patients to consult with pharmacists and receive medication-related advice without physically visiting a pharmacy [1].

The implementation of telepharmacy in emergency response has played a crucial role in improving the health awareness of the general public towards COVID-19 [2]. By leveraging telecommunications, telepharmacy has effectively disseminated information about the symptoms of the disease and measures to reduce its spread within the community. This has empowered individuals to make informed decisions about their health and take appropriate actions when necessary [3]. Moreover, telepharmacy has also been pivotal in referring individuals to appropriate healthcare facilities for testing if needed. This timely referral process ensures that individuals suspected of having COVID-19 can receive the necessary diagnostic screenings, thereby enabling early detection and intervention [4, 5].

Telepharmacy is especially beneficial for patients in rural areas who may have limited access to a nearby pharmacy [6]. It is also convenient for individuals with mobility issues or those who are unable to leave their homes. Additionally, telepharmacy can help reduce medication errors and improve patient adherence to prescribed treatments through its medication use review and medication adherence services [4]. By remotely reviewing a patient’s medication regimen, telepharmacy can identify any potential drug interactions or adverse effects, ensuring that patients are receiving the most appropriate and safe treatment. Furthermore, patient counseling services provided through telepharmacy allow individuals to have a thorough understanding of their medications and how to properly use them [5, 7].

Another advantage of telepharmacy is its ability to offer drug information services. Patients can access accurate and reliable information about their medications, potential side effects, and drug interactions, all from the comfort of their homes [8]. This not only increases patient knowledge and empowerment but also contributes to treatments through regular monitoring and counseling [7, 9]. Finally, telepharmacy can increase underserved populations’ access to medications and healthcare services [6].

Despite the potential advantages of telepharmacy, not much is known about how the general public feels about its use, despite the fact that numerous studies involving pharmacists and pharmacy students have been carried out [9–11]. In Malaysia, between 40 and 50% of community pharmacists expressed positive or somewhat positive views regarding the use of telepharmacy services. Those in the 20–39 age range were more likely to believe that telepharmacy is advantageous than those in the 40–59 age range [10]. In contrast, a significant proportion of pharmacy students in Malaysia, specifically 61%, exhibited favourable perceptions of telepharmacy services, while 67% and 68% demonstrated a high level of readiness and knowledge, respectively [11]. Notwithstanding this, the primary issues that were emphasised were inadequate cooperation with other healthcare units, insufficient understanding of telepharmacy among healthcare providers [10], excessive workload, and lack of incentives [11].

The next pivotal phase in maximising the utilisation of telepharmacy and resolving any potential obstacles or concerns is to ascertain the perceptions and knowledge of the general public in Malaysia regarding telepharmacy. For healthcare providers and policymakers, it is important to have a clear understanding of the general public’s perception and awareness of telepharmacy. This understanding can help inform decisions on how best to move forward with the implementation of telepharmacy and ensure that its benefits are fully realised. This study aims to assess the general public’s perception and awareness of telepharmacy, providing valuable insights for healthcare providers and policymakers in optimising its use and addressing any concerns or barriers that may exist.

## Materials and methods

A cross-sectional investigation was conducted using a convenient sampling technique from 30 Nov 2022 to 31 May 2023. Data collection was conducted through an online platform where the link for the survey was shared through social medias such as Whatsapp, Telegram, Facebook and Instagram. Consent from participants was obtained on the first page of the survey. The study received ethical approval from Research Ethics Committee, KPJ Healthcare University College (KPJUC/RMC/SOP/EC/2022/409) and Universiti Teknologi MARA Ethics Committee [REC/03/2023 (PG/MR/100)].

The total population of Malaysian aged 18 years and above was 23 million in 2022 [12]. With a significance level at α = 0.05 (two tailed) and confidence level of 95%, the estimated sample size was 385 [13]. A total of 387 adult Malaysians, aged 18 years and above, possessing proficiency in either Malay or English language, participated in this study. The data acquisition process was facilitated through Google Form, a software tool well-suited for survey data aggregation. The survey instrument comprised a total of 49 items, delineated into five distinct sections (Section A to E). The initial segment in the questionnaire (Section A) encompassed 11 items, delving into participant demographics and encompassing variables such as gender, age, ethnicity, educational attainment, occupational classification, monthly household income, current residence, prevalent medical conditions, frequency of medication usage, healthcare facility visitation frequency, and the underlying rationales prompting visits to community pharmacies.

In Section B, respondents were queried regarding their encounters with telepharmacy services. In Section C, the respondents’ digital readiness was evaluated in terms of their possession of the necessary knowledge and skills to engage in a telepharmacy session. The respondents’ perception of telepharmacy was evaluated in Section D whereas their willingness to adopt telepharmacy was assessed in Section E. Formulated as positively framed statements, responses were assessed on a 5-point Likert scale, ranging from 1 (strongly disagree) to 5 (strongly agree). The questionnaire’s construction encompassed both Malay and English languages. The content of the survey instrument was adapted and derived from Hossain et al. [14] and Peixoto et al. [15].

### Content validity

Face validity was evaluated by a group of distinguished professionals, including practitioner (n = 1), academician (n = 2), and members of the general public (n = 2). According to Taherdoost [16], this validation effort concentrated on evaluating the visual presentation of the questionnaire while taking into account elements like practicality, legibility, uniformity of style and layout, and lucidity of language expression. Deliberative and constructive observations were garnered and subsequently deliberated upon among the research team, precipitating necessary refinements. Prior to the start of the main study, significant changes, including terminological modifications, were made.

### Reliability test

The reliability assessment was conducted by administering questionnaires to a sample population consisting of 38 respondents. This evaluation encompassed two fundamental aspects: homogeneity and stability [17]. The determination of homogeneity, indicative of internal consistency for the scaled data, was computed utilising the Cronbach’s Alpha Test. Alpha values above 0.6 are considered acceptable [18]. The Cronbach’s Alpha reliability values for perception and awareness in this study were 0.908 and 0.971.

### Data analysis

Statistical Package for the Social Sciences (SPSS) software version 29 was employed for a comprehensive data evaluation. A descriptive analysis was conducted on the demographic characteristics of the respondents, which consists of frequencies, percentages and mean values.

## Results

### Demographic data

The demographic breakdown of the respondents is shown in Table 1. The respondents were mostly women (72.6%), with those between the ages of 18 and 27 making up the largest age group. Ethnically, the respondent pool was primarily composed of 89.4% Malay respondents. The majority of the respondents had an academic achievement equivalent to a bachelor’s degree. Employment categorisation demonstrated an approximate 1:1 ratio between those currently employed and those who were not. Notably, 31.8% reported an absence of income, while an equivalent percentage indicated an income below RM 4850. The residual 36.4% reported an income surpassing RM 4850. The majority, comprising 70.0%, indicated the absence of any prevalent medical conditions. On the other hand, the remaining respondents reported having at least one medical condition.

**Table 1:** Demographic data of the respondents (N = 387).

Demographics	N	%
**Gender** **Male** **Female**	106 281	27.4 72.6
**Age Group** **18–27 years** **28–37 years** **38–47 years** **48–57 years** **> 58 years**	224 68 60 19 16	57.9 17.6 15.5 4.9 4.1
**Race** **Malay** **Chinese** **Indian** **Others**	346 6 13 22	89.4 1.6 3.4 5.7
**Education Level** **Secondary** **Diploma** **Bachelor** **Masters or higher**	33 71 227 56	8.5 18.3 58.7 14.5
**Employment Type** **Employed Full Time** **Employed Part Time** **Unemployed** **Retired** **Student** **Housewife / Househusband** **Self Employed**	169 5 6 15 178 4 10	43.7 1.3 1.6 3.9 46.0 1.0 2.6
**Household Income (per month)** **No Income** **Below RM 4,850** **Between RM4,851—RM 10,970** **More than RM10,971**	123 123 102 39	31.8 31.8 26.4 10.1
**Diseases** **No** **Yes**	271 116	70.0 30.0
**Taking medications** **No** **Yes**	281 106	72.6 27.4
**No. of visit to hospital/primary clinic pharmacy for the last 6 months** **Never visited** **1–3 times** **4–6 times** **7–10 times**	157 195 25 10	40.6 50.4 6.5 2.6
**Reasons for visiting retail pharmacy** **To obtain over the counter medication** **To obtain health supplements** **To obtain prescribed medications** **To obtain home-diagnostic tools** **To ask for pharmacist advice** **To obtain surgical and home care** **To get general health information** **To get first aid information** **Others reason**	292 160 92 45 37 22 21 5 4	76.6 41.3 23.8 11.6 9.6 5.7 5.4 1.3 1.0

The majority of the respondents visited both hospital/primary clinic pharmacies and retail pharmacies with a frequency ranging between 1 to 3 occasions the last 6 months. Within this context, a noteworthy 76.6% of respondents resorted to retail pharmacies primarily for the acquisition of non-prescription medications, whereas 41.3% engaged in visits to procure health supplements. A mere 9.6% of the respondents availed themselves of pharmacist consultation services during their interactions with retail pharmacies.

Respondents were asked about their experience with telepharmacy. Of the 387 respondents, only 34 have participated in a telepharmacy service, giving a prevalence of 8.8%. Whereas 40.6% of the respondents have heard about telepharmacy services.

### Technology readiness

Table 2 shows the technology and digital readiness of the respondents. Almost all the respondents own a smartphone (99.2%). Only 0.8% do not own any personal digital device. More than 95% of those surveyed acknowledged being able to operate their technological device, access its camera, and know how to turn on both the camera and microphone. About 93.3% have access to a headset or headphones and stable internet connection. In addition to using their digital device for work-related tasks, sending and receiving emails, entertainment and social media, banking and electronic payments, online shopping, transportation, and navigation, over 80% of the respondents use it for voice, video, and text calls. Merely 53.0% of the respondents utilised health tracking apps on their gadget, and only 13.7% possess a wearable fitness tracker.

**Table 2:** Technology and digital readiness of the respondents (N = 387).

Questions	Yes	
	N	%
Do you have access to camera on your device?	378	97.7
Do you know how to enable the camera on your device and use it?	374	96.6
Do you know how to enable the microphone and use it to speak?	374	96.6
Do you know how to navigate your technological device?	372	96.1
Do you have a stable internet connection that would enable you to access telepharmacy services?	363	93.8
Do you have access to a headset or headphones?	361	93.3
Do you have access to following personal digital devices? Smartphone Laptop/desktop iPad Smartwatch Wearable fitness tracker (e.g. FitBit) I do not have access to any personal digital devices	384 298 165 78 53 3	99.2 77.0 42.6 20.2 13.7 0.8
What have you used your personal digital device(s) for? Telephone calls Texting/instant messaging Banking & electronic payments Social media / communication apps Sending & receiving emails Online shopping / ordering Work-related activities Video calling Entertainment Alarms & time management Transport & navigation Reading news Health tracking apps	378 362 348 348 347 341 341 338 334 320 301 244 205	97.7 93.5 89.9 89.9 89.7 88.1 88.1 87.3 86.3 82.7 77.8 63.0 53.0

### Awareness of telepharmacy

Based on Table 3, among the 387 respondents, more than 50% were aware that they can order medications from online pharmacies (AW6) (56.4%) and consult with pharmacists through online websites and mobile applications (AW5) (55.0%). Nonetheless, only 47.3% of the respondents knew what telepharmacy was (AW1). Less than 40% of respondents indicated they were unaware of telepharmacy services, which suggests a lack of understanding about them, their use, and their advantages. The mean score for two statements was less than three: query AW2: having enough information about telepharmacy (mean 2.8, SD 1.2) and AW3: having enough information about how to use telepharmacy services (mean 2.9, SD 1.2).

**Table 3:** Public awareness on telepharmacy (N = 387).

Item number:Awareness statements	Strongly Disagree/ Disagree		Neutral		Strongly Agree / Agree		Mean Score	SD
	N	%	N	%	N	%		
**AW1: I am aware of the term telepharmacy**	94	24.3	110	28.4	183	47.3	3.4	1.2
**AW2: In my opinion, I have enough information about telepharmacy**	148	38.3	144	37.2	95	24.5	2.8	1.2
**AW3: In my opinion, I have enough information about how to use telepharmacy services**	150	38.7	126	32.6	111	28.7	2.9	1.2
**AW4: In my opinion, I have enough information about the benefits of using telepharmacy services**	137	35.4	116	30.0	134	34.6	3.0	1.2
**AW5: I am aware that I can consult with pharmacists through online websites and mobile applications**	79	20.4	95	24.5	213	55.0	3.5	1.2
**AW6: I am aware that I can order medications from online pharmacies**	79	19.1	95	24.5	218	56.4	3.6	1.2

### Perceptions on telepharmacy

There were 21 perception-related items in the questionnaire, and the results are succinctly tabulated in Table 4. More than 80% of the respondents perceived that telepharmacy services are effective in saving time and effort (P2), would expedite the process of consultations (P11), have the potential to enhance the accessibility of the consultation process due to their streamlined mechanism (P8), provide patients with the opportunity to interact with pharmacists at their leisure, overcoming limitations related to time and location (P1), reduce service-related costs (P3) and create a quicker avenue for consultations (P11). Approximately 82.1% of the respondents believe that telepharmacy implementation is a good idea (P9) and will benefit them (P10), respectively.

**Table 4:** Public perceptions on telepharmacy (N = 387).

Item number: Perception statements	Strongly Disagree/ Disagree		Neutral		Strongly Agree / Agree		Mean score	SD
	N	%	%	N	%	N		
**P1: Telepharmacy service is important for patients to be able to communicate with pharmacists whenever and wherever they are**	13	3.4	58	15.0	316	81.7	4.3	.9
**P2: Telepharmacy services help to save time and energy**	7	1.8	39	10.1	341	88.1	4.4	.8
**P3: Telepharmacy services help to reduce service costs**	17	4.4	56	14.5	314	81.1	4.2	.9
**P4: Telepharmacy services will increase my compliance with medication**	22	5.7	118	30.5	247	63.8	3.9	1.0
**P5: Using telepharmacy services will improve my health.**	23	6	114	29.5	250	64.6	3.9	1.0
**P6: Using telepharmacy services will improve my knowledge of medication and disease**	13	3.4	65	16.8	309	79.9	4.2	.9
**P7: Using telepharmacy services will improve my medication management**	19	4.9	87	22.5	281	72.6	4.1	1.0
**P8: Using telepharmacy services will be easier for me to get a consultation from the pharmacist**	14	3.6	63	16.3	310	80.1	4.2	.9
**P9: Using telepharmacy services for accessing medical care is an appealing idea**	9	2.3	60	15.5	318	82.1	4.3	.8
**P10: In my opinion, telepharmacy services, such as online consultations and online drug delivery, will be useful for me**	13	3.3	56	14.5	318	82.1	4.23	.8
**P11: In my opinion, telepharmacy would enable consultations to be done much faster**	11	2.9	45	11.6	331	85.6	4.32	.8
**P12: In my opinion, telepharmacy services will be useful for follow-up medical consultations**	12	3.1	58	15.0	317	81.9	4.21	.9
**P13: In my opinion, telepharmacy services will be useful for emergency situations**	20	5.2	63	16.3	304	78.6	4.16	1.0
**P14: In my opinion, learning to use telepharmacy services is an easy matter**	18	4.7	95	24.5	274	70.8	3.95	.9
**P15: I think that using telepharmacy services will be simple**	17	4.4	84	21.7	286	73.9	3.99	.9
**P16: I think that telepharmacy services will be easily understandable and clear for me**	16	4.1	77	19.9	294	76.0	4.04	.9
**P17: I think that using telepharmacy services will be convenient**	17	4.4	80	20.7	290	74.9	4.05	.9
**P18: In my opinion, telepharmacy is accessible**	24	6.3	71	18.3	292	75.5	4.01	.9
**P19: In my opinion, online pharmacy services are more convenient than the services provided in physical retail pharmacy establishments**	36	9.3	119	30.7	232	59.9	3.76	1.0
**P20: I am confident that the pharmacist will be able to assess my condition via video conferencing as if I am at the pharmacy**	65	16.8	115	29.7	207	53.5	3.57	1.1
**P21: The lack of physical contact in a video visit is NOT a problem for managing my disease**	48	12.4	122	31.5	217	56.0	3.65	1.1

Respondents agreed that telepharmacy could improve medication adherence (P4) (63.8%) and improve their overall health by helping them learn more about their illnesses and medications (P6) (79.9%), and make it easier to stick to their medication schedule (P7)(72.6%). Additionally, the general public belief that telepharmacy services would be beneficial and valuable in the context of post-treatment medical consultations (P12) (81.9%) and emergency situations (P13) (78.6%).

Between 70 and 76% of the respondents thought that telepharmacy was convenient (P17), it is easy to learn to use telepharmacy services (P14), as it was simple (P15), accessible (P18) and easily understandable and clear (P16). However, only 59.9% of respondents thought that services offered by online pharmacies were more convenient than those offered by physical locations (P19).

Looking at the pharmacist dimension in the survey, 53.5% of respondents thought that the pharmacist could conduct a comprehensive evaluation via video conference, mimicking an in-person interaction (P20). Furthermore, 56.0% of respondents thought that if there was no physical contact, a video consultation would still be just as successful in managing their health condition (P21).

### Willingness towards telepharmacy

A considerable portion of respondents (55.0%) expressed a preference for in-person visits to retail pharmacies (Table 5). On the other hand, only 25.6% of respondents said they preferred phone calls, videos, or online or application consultations, and 14.7% said they had no preference.

**Table 5:** Public preference on the mode of pharmacists’ consultation (N = 387).

Statements	N	%
**W1 What will be your preference if you need a pharmacist consultation?** ** I prefer an in-person visit to a retail pharmacy** ** I have no preference or equal preference** ** I would prefer to use an online/app-based tool** ** I would prefer to have a consultation over the phone** ** I do not know** ** I prefer a video visit**	213 57 53 31 18 15	55.0 14.7 13.7 8.0 4.7 3.9

Table 6 presents the responses regarding willingness. Only 30.2% agreed they are willing to pay for telepharmacy services while 48.1% agreed that they will use telepharmacy services to manage their medicine and disease. For W4, 65.6% were more likely to use telepharmacy services to meet their future need of health. 49.1% will be more likely to use telepharmacy services offered by the independent retail pharmacy, 59.9% will use telepharmacy at an established chain retail pharmacy and 67.5% agreed that they will be more likely to use telepharmacy services if government agencies offer it. For W8, 70.8% willing to share their health data with healthcare providers.

**Table 6:** Public willingness towards telepharmacy (N = 387).

Willingness (W)	Strongly Disagree / Disagree		Neutral		Strongly Agree / Agree		Mean Score	SD
	N	%	N	%	N	%		
W2 I am willing to pay for telepharmacy services	92	23.8	178	46.0	117	30.2	3.6	1.0
W3 I am willing to use telepharmacy services to manage my medicine and disease	34	8.7	167	43.2	186	48.1	3.8	0.9
W4 I will more likely use telepharmacy services to meet the future needs of my health	18	4.7	115	29.7	254	65.6	3.5	1.0
W5 I will more likely use telepharmacy services offered by the independent retail pharmacy	48	12.4	149	38.5	190	49.1	3.7	1.0
W6 I will more likely use telepharmacy services if known chain retail pharmacy establishments, would offer them	36	9.3	119	30.7	232	59.9	3.9	1.0
W7 I will more likely use telepharmacy services if government agencies such as the government hospital and health clinics offer it	24	6.2	102	26.4	261	67.5	3.9	0.9
W8 I am keen to share my health data with healthcare providers via telepharmacy	21	5.5	92	23.8	274	70.8	3.1	1.1

## Discussion

The current research aimed to investigate the public’s comprehension and viewpoints regarding telepharmacy, focusing on their awareness of the concept and their inclinations toward its integration. A comprehensive analysis of respondents’ technological readiness highlighted a significant proficiency in digital capabilities, evidenced by widespread smartphone ownership and adeptness in utilising diverse features such as camera and microphone functionalities, internet stability, and familiarity with digital tools. Moreover, a survey conducted by the Malaysian Communications and Multimedia Commission in 2020 [19], reaffirmed the prevalence of smartphone ownership among Malaysians.

Digital literacy encompasses “the confident and critical use of a full range of digital technologies for information, communication and basic problem-solving in all aspects of life”[20]. In order for digital healthcare services to be delivered effectively, users must possess digital literacy, which entails being adept at utilising technology [21]. The possession of appropriate technological tools and proficiency in utilising digital platforms empower individuals to actively participate in telepharmacy services and obtain maximum advantages from them. This enhances user engagement with digital services and helps bridge healthcare access gaps, especially where traditional infrastructure is lacking. Nonetheless, the findings pertaining to survey respondents’ limited engagement with wearable fitness trackers and health-tracking applications raise pertinent considerations regarding their utilisation of technology for health-related purposes. A striking divergence in usage patterns was evident when compared with a comprehensive survey conducted in the United States, encompassing 1604 mobile users, wherein a majority had embraced health-related applications, primarily centered around fitness and nutrition [22].

Interestingly, those individuals who stand to derive the greatest advantages from health apps—particularly those reporting low levels of physical activity and suboptimal self-reported health—exhibited a marked reluctance in adopting and utilising these technological tools [23]. This intriguing contradiction strongly suggests the existence of barriers hindering specific segments of the population from accessing and employing health apps for example, concerns about privacy and security, skepticism over context sensing, the effort required for consistent use, social stigma, and the necessity for efficiency and convenience [24].

This notable disparity between the potential beneficiaries of health technology and their actual utilisation underscores the pressing need to uncover and address the impediments preventing certain demographics from engaging with these resources effectively. To effectively address these barriers, strategies should focus on enhancing user-friendliness, ensuring credibility, addressing privacy concerns, optimising efficiency, and allowing personalisation and control [23]. Further empirical investigation is imperative to delve into these barriers comprehensively. Insights garnered from such research endeavors will be pivotal in formulating targeted strategies aimed at ensuring equitable access and utilisation of technology, especially among those individuals who stand to gain the most from these health-centric innovations. Such efforts will be instrumental in fostering inclusive health technology ecosystems that cater to the diverse needs of varied population segments, ultimately enhancing health outcomes and wellbeing on a broader scale.

The study uncovered a spectrum of awareness levels concerning telepharmacy services among respondents. A noteworthy portion demonstrated familiarity with capabilities such as medication pre-ordering and online consultations with pharmacists. However, the study revealed a striking revelation concerning the fundamental understanding of the telepharmacy concept, as evidenced by low average scores on pertinent statements. These echoes findings observed in Jordan, a region where telepharmacy remains relatively new, similar to Malaysia. Among 800 respondents, a mere 16% had utilised the service, while a staggering 43% were unaware of its existence [25].

Notably, despite the longstanding existence of telemedicine, telehealth, and telepharmacy, it appears that a considerable segment of the population remains uninformed about these services and their implications [6] which can result in underutilisation, limited access to care, poorer health outcomes, and increased healthcare disparities [1]. The rapid adoption and realisation of the potential of telehealth and telepharmacy were propelled by the pivotal moment marked by the emergence of the COVID-19 pandemic. [2]. The crisis compelled healthcare systems to swiftly pivot and incorporate these services, ensuring continued access to essential care. The expeditious implementation during this period underscored the feasibility and benefits of telepharmacy services [2, 4].

This transition illuminated the viability and advantages of telepharmacy, signaling the appropriateness of sustained provision beyond the pandemic era and highlighted the indispensable role of telehealth and telepharmacy in ensuring healthcare accessibility and continuity, warranting their continued integration into standard healthcare frameworks. Such continuity stands poised to further enhance healthcare delivery and accessibility, transcending the confines of emergency response to become integral components of future healthcare landscapes.

Our findings showed that the Malaysian public generally exhibits a favorable awareness, perceptions, and willingness towards the implementation of telepharmacy, as evidenced by a mean score exceeding 3 for the majority of questions across these three domains. The perception of telepharmacy services among respondents revealed predominantly positive attitudes, emphasizing its potential to save time, enhance accessibility, and streamline the consultation process. The belief in its effectiveness in improving medication- and disease-related knowledge, facilitating post-treatment consultations, and being valuable in emergency situations was notably high. On the contrary, the willingness domain depicted a mixed sentiment, with a substantial preference for in-person visits over telepharmacy consultations.

A study in Saudia Arabia indicated that 83% of the public surveyed held a favorable view of telepharmacy services [26], and similarly, respondents in Jordan were found to have a positive perception of telemedicine implementation [25]. These findings suggest that the acceptance and perception of telepharmacy may vary across different cultural contexts for example, the accessibility of internet and the level of digital literacy among the population can significantly impact the acceptance of telepharmacy services. It is important for future research to explore the underlying factors that contribute to these differing attitudes and levels of engagement in order to effectively implement telepharmacy services worldwide.

It is unwarranted to emphasise that over 55% of the respondents continued to favor visiting a retail pharmacy in person. In a similar vein, 64% of patients in Indonesia indicated a preference for in-office consultations during their medical appointments [27]. These findings suggest that there may be cultural and societal factors at play that influence individuals’ preferences for in-person interactions in healthcare settings.

Communication with healthcare professionals (HCP) does not need to be entirely virtual or online, adaptations can be made so that a rapport is built with patients in the first few visits, which would ease the subsequent virtual consultation. HCPs can build rapport with patients by showing positive attitudes and cultural competence, employing effective verbal and nonverbal communication, fostering strong relational connections, and maintaining a professional and private environment. This can help establish trust and a sense of comfort, which may be crucial for effective virtual consultations [28].

Combining in-person meetings with online consultations is a wise approach to maximise telepharmacy’s effectiveness while also accommodating patient preferences. This approach could strike a balance by providing personalised care options, educating patients, cultivating interpersonal connections, ensuring seamless integration and accessibility, addressing privacy concerns, and continuously evaluating and adapting services to patient needs and preferences, thus, improving healthcare delivery and accessibility for specific patient demographics and acknowledging and respecting the diverse inclinations of patients. Therefore, it is important for healthcare providers to consider these factors and provide options for both in-person and virtual consultations to accommodate the preferences of patients while still leveraging the benefits of telepharmacy in improving access and convenience for certain patient populations.

It is important to note that, while telepharmacy offers significant advantages, its use may present risk due to the lack of direct HCP-patient interaction, compared to traditional settings. This absence can heighten the risk of issues such as polypharmacy, leading to increased hospitalizations, deteriorating outcomes, and rising healthcare costs [29].

One potential drawback of this research is its utilisation of convenience sampling via an online survey. It is important to note that this approach may have excluded people who do not participate in or are unfamiliar with online surveys, potentially excluding a large portion of the population who does not use digital technologies or social media, given that the survey was disseminated through social media platforms. Specifically, as participants were not randomly selected, the demographic composition in this study may not entirely reflect the general population of Malaysia, which consists of 50.3% males and 49.7% females, with a broader age distribution and ethnic diversity [12]. This could affect the extent to which our results can be applied to different groups or settings. Readers should interpret the results with caution, considering the potential biases arising from the sampling method used.

## Conclusion

The study conducted in Malaysia aimed to gauge public attitudes toward telepharmacy, revealing a society well-versed in digital tools due to widespread smartphone ownership. However, it highlighted a noteworthy disconnect in the utilization of health-tracking technology despite this digital readiness. The research uncovered varying levels of awareness about telepharmacy services, notably showcasing a lack of understanding about its fundamental concept among respondents. Despite positive perceptions regarding its benefits in saving time, enhancing accessibility, and streamlining consultations, only 48.1% expressed a willingness to adopt telepharmacy in the future, indicating hesitancy or reservations.

To address this knowledge gap, the study suggested implementing targeted educational initiatives to bolster understanding and potentially improve acceptance of telepharmacy, aiming to enhance patient outcomes by improving access to pharmaceutical care and medication management, especially during crises like the COVID-19 pandemic. Moving forward, research efforts should focus on crafting strategic integration methods for telepharmacy within Malaysia’s healthcare systems, taking into account the preferences and concerns highlighted by survey respondents. Additionally, conducting longitudinal studies to assess the real-world implementation and outcomes of telepharmacy interventions across diverse demographic groups would provide invaluable insights into its efficacy and acceptance, crucial for optimising its role in Malaysia’s healthcare landscape. While our study provides valuable insights, it is important to acknowledge the limitations associated with the use of convenience sampling which may introduce bias and limit the generalizability of our findings. Future research should consider employing more rigorous sampling techniques to enhance the generalisability of the findings.

## Supporting information

S1 Checklist (DOCX)
